# Occurrence of total aflatoxins (B1, B2, G1, G2) in commercial peanut and peanut butter from the Saudi market in the period from 2015 to 2020

**DOI:** 10.1016/j.toxrep.2024.101797

**Published:** 2024-11-18

**Authors:** Abdullah Al tamim, Rawan Alharbi, Zeyad Aldosari, Yaser Almasoud, Abdullah A. Al Sayari, Mohammed A. Almutairi, Abdullah M. Alowaifeer, Hibah Alharbi

**Affiliations:** Saudi Food & Drug Authority (SFDA), Riyadh 11561, Saudi Arabia

**Keywords:** Mycotoxin, Aflatoxins B1 B2 G1 G2, Peanut, Peanut butter

## Abstract

Nut products are susceptible to contamination with mycotoxin, especially aflatoxins, which results of mold growth during harvest or storage. This study aimed to evaluate the occurrence of aflatoxins in peanut products from the Saudi market. A total of 472 samples of peanut and peanut butter imported from various countries were collected in the period from 2015 to 2020. All samples were analyzed by High-Performance Liquid Chromatography (HPLC). Total aflatoxins (B1, B2, G1, G2) were found to exceed the maximum level (ML) in 38 samples (8.1 %) with a concentration range of 15.56–973.21 µg/kg, while 434 samples were below ML (91.9 %). Specifically, in Peanut butter samples, 27 out of 333 were contaminated with Aflatoxins (8.8 %), and 11 out of 139 peanut samples were contaminated with aflatoxins (8.6 %). The results show that the percentage of Aflatoxins contamination was reduced in the period (2019 – 2020) compared to (2015 – 2018), due to the control of mycotoxins in Saudi Arabia which played an important role in reducing the contamination of AFs in peanut products.

## Highlights

• Studies indicate a high level of AFs contamination in peanut and peanut butter samples collected at the border, highlighting a significant health risk associated with these products.
• The study reveals a decrease in AFs contamination from 2019 to 2020 compared to the period from 2015 to 2018. This reduction is attributed to effective mycotoxin control measures implemented in Saudi Arabia.
• The findings emphasize the necessity of implementing AFs control strategies to mitigate health risks posed by AFs contamination in peanut products.
• Increasing community awareness about the risks of AFs and educating on methods to reduce AFs formation is crucial in addressing the issue of contamination in peanut products.

## Graphical Abstract

**Figure:**
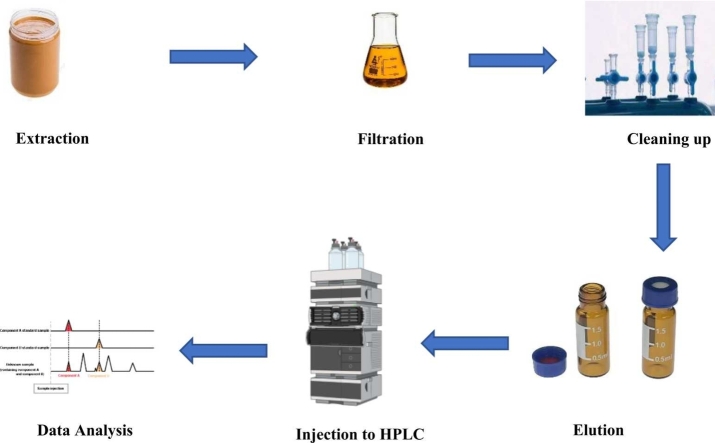

## 1 Introduction

Mycotoxins are compounds with low molecular weight produced by filamentous fungi belonging to the *fusarium, penicillium*, and *aspergillus* genera on different types of agricultural products [31]. *Aspergillus* is the most common mycotoxin producing fungi and is responsible of generating different life-threatening mycotoxins, which include but not limit to aflatoxins, ochratoxins, cyclopiazonic, aflatrem, terrein, gliotoxin, citrinin sterigmatocystin, and patulin [23], [25]. Fungal growth, as well as mycotoxin secretion, could happen at any stage of crop production depending on environmental factors such as humidity, water activity, temperature, and farming practices (cropping, harvesting, and storage conditions) [34]. It has been observed that the optimum temperatures for aflatoxins production is at 30 °C. Unfortunately, eliminating contamination with these toxins during food industrial processing remains an obstacle because of the stability at high temperatures and cannot be destroyed by heat treatment. Hence, these toxins can be found in infant food, children and bakery products [3].

Crops contamination with Mycotoxins is a serious threat to public health; therefor, mycotoxins have attracted the attention of human, animal, and plant health experts [26], [29]. The Food and Agriculture Organization (FAO) estimates that current mycotoxin occurrence is above the Codex and EU limits by 25 % [14]. World Health Organization (WHO) has tries to reduce the worldwide problem of mycotoxin contamination in food chains by implementing regulatory guidelines. Additionally, the joint scientific advisory committee stated the responsibility for the evaluation of health risks from mycotoxins [8], [15], [29], [31].

Mycotoxins contamination on agricultural products does not only impact humans and animal health, but also trade and food security. In the case of the United States, the loss of maize due to mycotoxins costs farmers around $160 million, whereas in Africa it is reported mycotoxin contamination cause a loss of $450 million [16], [35].

The most common mycotoxins are aflatoxins, which include four compounds: Aflatoxin B1, Aflatoxin B2, Aflatoxin G1, and Aflatoxin G2 [23]. Aflatoxins are usually found in agricultural products that are grown in warm climates such as maize, cereals, and peanuts [6]. Aflatoxin growth and invasion of agricultural products is a long biochemical process that starts with the growth of the fungi then subsequent the production of the toxin [2]. Aflatoxin synthesis consists of 13 enzymatic reactions starting with the conversion of acetate to norsolorinic acid. As many as 30 genes are involved in the fungal production of aflatoxins [36]. Not all Aspergillus species generate aflatoxin and not all species invade agricultural products. In addition, the severity of aflatoxin contamination of agricultural products is determined via fungal ecology [10], [30].

According to International Agency for Research on Cancer (IARC), Aflatoxin B1 classified as a group 1 carcinogen because of it is toxicity, mutagenic, immunotoxin, teratogenic, and carcinogenic effect on humans and animals [24]. Studies indicated that Aflatoxin G1 is the most common aflatoxins than Aflatoxin B2 and Aflatoxin G2 in aflatoxins contamination outbreaks. Moreover, Aflatoxin B1 show higher carcinogenicity as well as lethal followed by Aflatoxin G1, Aflatoxin B2, and Aflatoxin G2. Laboratory mice Aflatoxin G1 orally given Aflatoxin G1 have displayed carcinomas, renal-cell tumors of the kidney cells, increase in triglycerides, and cells autolysis [21].

Several acute aflatoxins exposures (aflatoxicosis) cases have been reported. In Kenya, an outbreak aflatoxicosis fatality reached 40 %, while Tanzania documented to be as high as 30 % [7], [20]. It has been reported the outbreak of aflatoxins in India and stands for the 30 % of food contamination globally [23]. In Cases of the population with gastric carcinoma and esophageal carcinoma, a great amount of Aflatoxin G1 was found in China [23].

Since the discovery of aflatoxins and their effect, countries rushed to act and minimize their impact. Codex standard No. (CAC 193) and the European Union (EU) have set the ML of total Aflatoxin in peanut products at 20 µg/kg. The Gulf Cooperation Council, which includes Saudi Arabia, Bahrain, Kuwait, Oman, Qatar, and United Arab Emirates have set regulations for total Aflatoxin in food has set the ML at 20 µg/kg [4]. In 2019, the Saudi Food and Drug Authority (SFDA) has updated its ML for peanut products to 15 μg/kg [28].

In this study, the occurrence of aflatoxins in peanut products marketed in Saudi Arabia in the period from 2015 to 2020 was evaluated. The results reported in this manuscript are a part of the Saudi Food and Drug Authority’s compliance program.

## 2 Materials and methods

### 2.1 Sample collection

A total 472 different peanut samples (139 of peanut and 333 of peanut butter) were collected from different boarders as part of SFDA’s compliance programe. To overcome irregular mycotoxins distribution, authorized and trained SFDA inspectors collected the samples according to CODEX Alimentarius Commission general guidelines on sampling (FAO/WHO, 2004) [19]. Aggregate samples of approximately >1 kg per sample were transported to SFDA laboratory campuses (Riyadh, Jeddah, and Dammam – Saudi Arabia) in dry suitable containers immediately after collection. At the laboratory, each sample was physical checked for deformities before homogenizing the samples for extraction.

### 2.2 Chemicals and reagents

Total aflatoxins certified reference standard was purchased from Trilogy® (TSL-108–10 lot:200723–041) with a concentration of 10.0 μg/ML in acetonitrile. High-performance liquid chromatography (HPLC) grade methanol (CAS No. 67–56–1), and acetonitrile (CAS No. 75–05–8) was acquired from Supelco, Merch, Darmstach - Germany. Ultrapure water was obtained from an Milli-Q® Direct Water Purification System. Sodium chloride was acquired from NENTECH, UK.

### 2.3 Extraction and clean up

The method of extraction was conducted based on ISO 16050:2003 [1]. Twenty-five g of test sample was homogenized in a blender jar with 5 g of sodium chloride and 125 ML (methanol 7: water 3) of extraction solvent. Homogenization was conducted using blender jar for 2 min at high speed. Afterwards, the mixture was filtered through a fluted filter paper, then15 ML of the filtrate was pulled with a pipette and placed into a conical flask with glass stopper. Add 30 ML of water, stopper the flask and mix. Then, second diluted extract 45 ML was filtered through a microfiber filter paper. The immune-affinity column was tempered to ambient temperature and firmly attached to a syringe barrel, and placed on a column rack. Fifteen ML of the extract was passed through the column. The column was washed with 20 ML of distilled water. To elute bounded aflatoxin of the column, 1 ML of methanol: 1 ML of distilled water 1/1 (v/v) mixture was passed through the immune-affinity column. After that, transferred to a HPLC vial.

### 2.4 Liquid Chromatography with FLD Analysis

**Fig. 1:**
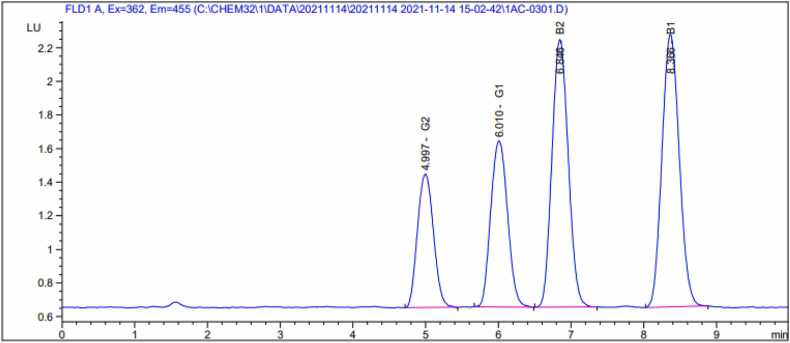
The chromatogram of separated to total Aflatoxin by HPLC-FLD.

An Agilent 1200 Series HPLC containing autosampler, degasser, fluorescence detector, binary pump, derivatization pump and column compartment was used to analyze the samples. Total Aflatoxins were analyzed using Phenomenox, Kinetex -C18 (2.6 µ; 100 mm length x 3.00 mm dia) under isocratic condition (mobile phase A: acetonitrile (1.75 %) methanol (1.75 %) deionized water (6.5 %)). The flow rate was 1.0 ML/min, the column temperature was set at 60 ºC, and the injection volume was 75 µL. The excitation wavelength was 362 nm and the emission wavelength was 455 nm. The retention time of total aflatoxins were determined to be between 4.00 min to 9.00 min (Fig. 1). Standards at the following levels: 0, 6.25, 12.5, 25, 37.5 µg/L to generate a calibration curve (Table 1).

**Table 1:** Recoveries, LOD, LOQ and linearity for the analysis of Total Aflatoxins in Peanut.

**Analyte**	**Mean recoveries (%)**	**RSD%**	**LOD (µg/Kg)**	**LOQ (µg/Kg)**	**Linearity**
**Aflatoxin B1**	107	5.23	0.38	1.26	r2 = 0.999
**Aflatoxin B2**	105	5.57	0.10	0.33	r2 = 0.999
**Aflatoxin G1**	108	5.68	0.20	0.69	r2 = 0.999
**Aflatoxin G2**	83	7.25	0.10	0.33	r2 = 0.999
**Total Aflatoxin**	104	5.51	0.77	2.59	r2 = 0.999

### 2.5 Quality control

Spiked samples were prepared and analyzed with each sample batch to ensure accuracy and reliability. The recovery range of total aflatoxin in the spiked samples was found to be between 70 % and 120 %, and the expanded uncertainties of all measured compounds were ≤ 20 % which was calculated based on the (1), (2). Blank samples were also analyzed to confirm the absence interferences. For the calibration curves, the minimum acceptable correlation coefficient (r2) was set at >0.9995 for a minimum of 3 levels. Moreover, the limit of quantification (LOD) was determined as 3 times the standard deviation of lowest level, while the limit of quantification (LOQ) was determined as 10 times the standard deviation of lowest level. All laboratory performing the analysis are accredited under ISO 17025 for the determination of aflatoxins B1, B2, G1and G2 in peanut using HPLC-FLD.

(1) $$\mathrm{The standard uncertainty}=\sqrt{{\left(\mathrm{precision uncertainty}\right)}^{2}+{\left(\mathrm{bias uncertainty}\;\right)}^{2}}$$

(2) The expanded uncertainty=2×the standard uncertainty

## 3 Result and discussion

**Fig. 2:**
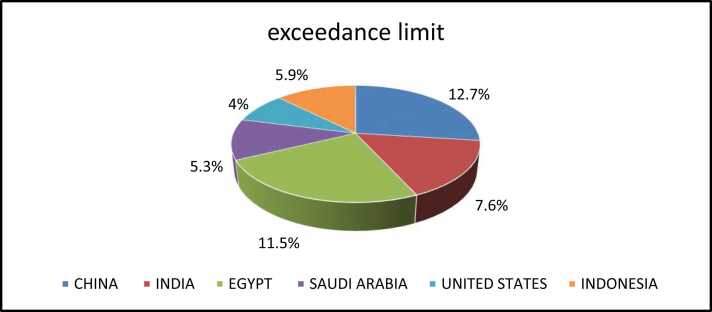
The percentage of exceedance limit of the samples from different countries;2015–2020.

**Fig. 3:**
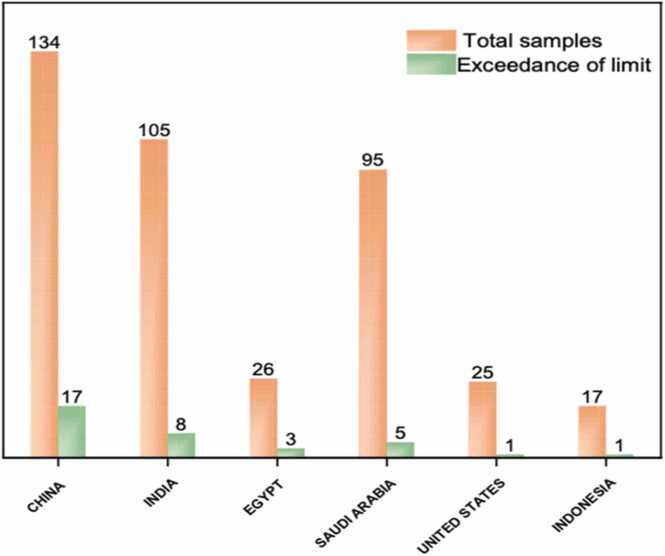
**.** the exceedance of limit and total samples from 2015 to 2020.

The analyzed samples of peanut and peanut butter originated from different countries. The majority of the samples (85.2 %) were from China, India, Egypt, Saudi Arabia, and Indonesia. The total aflatoxins (B1, B2, G1, G2) samples that exceeded the maximum level ML were 38 samples (8.1 %) out of 472 samples with the concentration range of 15.65–973.21 µg/Kg, while the rest of samples were below ML with the concentration range of 2.69–14.43 µg/kg. Furthermore, in peanut butter samples, 27 out of 333 were contaminated with AFs (8.8 %), and 11 out of 139 peanut samples were contaminated with aflatoxins (8.6 %) (Table 2). Moreover, the number of contaminated samples from China were 17 out of 134 (12.7 %), Egypt 3 out of 26 (11.5 %), India 8 out of 105(7.6 %)], Indonesia 1 out of 17 (5.9 %), Saudi Arabia 5 out of 95 (5.3 %), and United States 1 out of 25 (4 %). (Fig. 2), (Fig. 3). The highest recorded level was 973.21 μg/kg of total aflatoxins (B1, B2, G1, G2) in a sample from India. All samples that exceeded the ML of total aflatoxins were rejected by Saudi Food and Drug Authority (SFDA) at the border. The percentages of the samples exceeded allowable limit or contaminated were measured as per the Eq. (3).

(3) $$\mathrm{The percentages of the samples exceeded allowable limit or contaminated}=\left(\frac{\mathrm{number of the samples exceeded allowable limit or contaminated}}{\mathrm{the total number of the}\mathrm{targeted}\mathrm{samples}}\right)\times 100$$

**Table 2:** The data of total aflatoxins in peanuts and peanuts butter samples from 2015 to 2020.

Year/ Type	Concentration Range (µg/kg)	Exceedance of ML	Within Limit	Grand Total	% Within Limit	% Exceedance SFDA ML	Exceedance SFDA ML
2015		14	93	107	86.9	13.1	20
Peanut butter	2.8–11.39		12	12	100	0.0	20
Peanuts	2.75–816.47	14	81	95	85.3	14.7	20
2016		2	32	34	94	6	20
Peanut butter	3.59–5.85		7	7	100	0	20
Peanuts	3.79–50.82	2	25	27	92.6	7.4	20
2017		1	18	19	94.7	5.3	20
Peanut butter	7.78–50.3	1	5	6	83.3	16.7	20
Peanuts	0–16		13	13	100	0	20
2018		5	100	105	95.2	4.8	20
Peanut butter	5.68–6.3		11	11	100	0.0	20
Peanuts	3.24–973.21	5	89	94	94.4	5.3	20
2019		9	110	119	92.4	8	15
Peanut butter	2.74–47.22	6	38	44	86.4	13.6	15
Peanut	4.66–868	3	72	75	96	4	15
2020		4	84	88	95.5	4.5	15
Peanut butter	2.69–53.1	4	55	59	93.2	7	15
Peanut	0		29	29	100	0	15
**Grand Total**		**38**	**434**	**472**	**92**	**8.1**

**Table 3:** A survey of peanut and peanut butter contaminated with aflatoxins.

Country	Food matrices	Sample size	Concentration range (ug/kg)	Reference
Gambia	peanut	1168	112–8.55	(E. [18]
Zambia	Raw peanut	92	48.67 –0.014	[9]
Haiti	Peanut	21	787–2.0	[27]
	Peanut butter	32	2720–2.0	
Saudi Arabia	peanut	5	90–11	[11]
Pakistan	Peanut with shell	10	14.5–1.5	[22]
	Peanut without shell	10	12.8–0.7	
Sudan	Peanut butter	43	853–26.7	[13]
China	peanut	2983	113.50–0.16	[12]

**Fig. 4:**
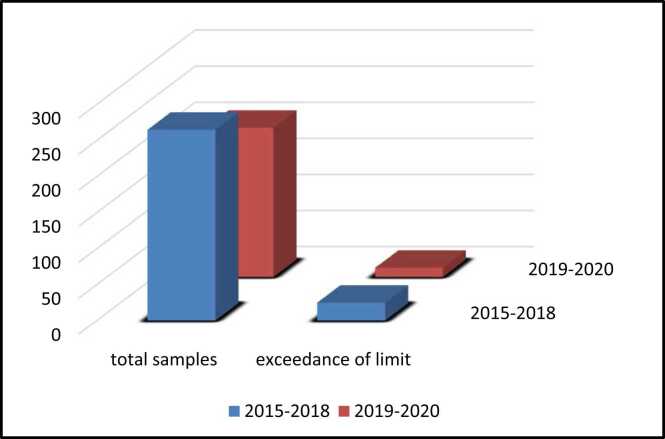
Comparing the exceedance of limit samples from 2015 to 2018 and 2019–2020.

In 2019, a new regulation has been enforced by SFDA to protect the consumers from any possible food safety issue. The new regulation includes changing in the ML of total aflatoxin for peanut and its products from 20 µg/kg to 15 µg/kg. The data from 2015 to 2018 shows that 22 out of 265 total samples were contaminated by AFs with 8.3 % rejection rate. In another hand, from 2019 to 2020 13 out of 207 total samples were contaminated by Aflatoxins and exceeded ML with rejection rate 6.3 %, which is a lower percentage than the previous years (Fig. 4). More importantly, this result proves the efficacy of the well-advised regulation of SFDA. Moreover, it was proposed to lower the ML of total aflatoxin in ready-to-eat peanut to 10 µg/kg; therefore, if the proposed regulation were applied in our data, the rejection rate would be 9.5 % which may arise further trade impediments for the exporter countries.

Our findings in this study were found to be in agreement with most published data from different countries. A study of aflatoxin contamination in Gambian peanuts in the period between 2011 and 2018 discovered that 42 % of 1168 examined samples had levels of aflatoxin higher than the Codex ML, with the highest mean value been detected in 2011 (112 μg/kg), and the lowest mean of 8.55 μg/kg in 2018(E. [18]. In Zambia, 51 samples of a total of 92 peanuts samples were found to be positive for AFs in with the range of 0.014–48.67 μg/kg; however, only 12 % were over the EU ML[9]. Another research done in Haiti between 2012 and 2013 found that raw peanuts and peanut butter were positive for AFs at 14 % and 97 %, respectively [27]. In survey carried out in 2009 in Qassim region, Saudi Arabia 80 % of peanuts samples were found to be positive for AFs with a concentration range of 11–90 μg/kg [11]. Moreover, a study of post-harvest peanuts collected in China between 2009 and 2014 from 112 countries, 8.55 % of 2983 samples exceeded the Chinese ML (<20 μg/kg) [12]. In Sudan, researchers found that all 43 samples of peanut butter were contaminated with AFs [13].

Also, a distinctive study was conducted to find out the effect of peanut shells on the presence of mycotoxins. The presence of peanut shells has a slight effect of the occurrence of aflatoxins. In the report of Luttfullah and Hussain, AFs positive in Pakistan peanuts with a shell by 40 % while in peanuts without a shell by 50 % [22].

The results of previous studies were not expected due to the presence of mycotoxins in large proportions in peanut products. Hence, many regulatory bodies and organizations including The American Peanut Council (APC) provide excellent Good Manufacturing Practices for Peanut Product Manufacturers, which are assessed to minimize the hazard of nuts toxins that include but do not limit the moisture control, processing control, hygiene, and storage [32].

Some socioeconomic factors, such as informal marketing systems, insufficient transportation modes, lack of needed equipment, lack of information and knowledge on appropriate pre-and postharvest management, and poor governmental regulations and legislation, can contribute to the increase of aflatoxins contamination [33]. Additionally, one of the useful tools to prevent human exposure to AFs is raising awareness of the aflatoxins-related risk (A. [17].

In Saudi Arabia, peanuts and peanut butter are imported from various countries. However, the contamination of peanut products could have occurred during importation or prior to that. This kind of food products are very sensitive and could be contaminated with AFs during harvest, treatment, transportation or storage. The random tropical conditions including high temperatures, moisture, unseasonal rainfalls, monsoons, and floods help to fungal growth of AFs [5]. Up to this point, it is difficult to monitor all the food chain across the world, but the advice is to minimize the importation from countries where the majority of the samples were contaminated with the Aflatoxins. Furthermore, when purchasing peanut products from stores, consumers should be aware that storage should be in good conditions (at low humidity and low temperatures), according to the label on the product to prevent the formation of Aflatoxins [32].

## 4 Conclusion

This study evaluates the occurrence of AFs in peanut and peanut butter at the border.

Based on a total of 472 samples collected from different countries from 2015 to 2020. As seen above, most studies reported that the level of AFs contamination in peanut products is high and poses a significant threat to health. Moreover, AFs control strategies are required to avoid health risks caused by AFs contamination. Our result shows that the percentage of AFs contamination was reduce in the period (2019 – 2020) compared to (2015 – 2018), due to the control of mycotoxins in Saudi Arabia which played an important role in reducing the contamination of AFs in peanut products. Thus, community awareness of the risk of AFs, also the potential ways to reduce its formation should be increased.

## Author statement

We confirm that the manuscript has been read and approved by all named authors and that there are no other persons who satisfied the criteria for authorship but are not listed. We further confirm that the order of authors listed in the manuscript has been approved by all of us.

## CRediT authorship contribution statement

**Rawan Alharbi:** Writing – original draft, Validation, Formal analysis, Conceptualization. **Mohammed A. Almutairi:** Writing – original draft, Supervision, Methodology. **Abdullah A. Al Sayari:** Writing – review & editing, Methodology. **Yaser Almasoud:** Writing – review & editing, Supervision, Data curation. **Zeyad Aldosari:** Methodology, Investigation, Formal analysis, Data curation. **Hibah Alharbi:** Writing – review & editing, Visualization. **Abdullah M. Alowaifeer:** Visualization, Validation.

## Declaration of Competing Interest

The authors’ affiliations are as shown on the cover page. The authors have sole responsibility for the writing and content of the paper.

## Data Availability

Data will be made available on request.

## References

[1] 16050, & I S O. (2003). *Foodstuffs–Determination of Aflatoxin B1, and the Total Content of Aflatoxins B1, B2, G1 and G2 in Cereals, Nuts and Derived Products–High-Performance Liquid Chromatographic Method*.

[2] Abrar M., Anjum F.M., Butt M.S., Pasha I., Randhawa M.A., Saeed F., Waqas K. (2013). Aflatoxins: biosynthesis, occurrence, toxicity, and remedies. Crit. Rev. Food Sci. Nutr..

[3] Ahlberg S., Randolph D., Okoth S., Lindahl J. (2019). Aflatoxin binders in foods for human consumption—can this be promoted safely and ethically?. Toxins.

[4] Al-Jaal B., Salama S., Al-Qasmi N., Jaganjac M. (2019). Mycotoxin contamination of food and feed in the Gulf Cooperation Council countries and its detection. Toxicon.

[5] AlFaris N.A., ALTamimi J.Z., ALOthman Z.A., Al Qahtani S.F., Wabaidur S.M., Ghfar A.A., Aldayel T.S. (2020). Analysis of aflatoxins in foods retailed in Saudi Arabia using immunoaffinity column cleanup and high-performance liquid chromatography-fluorescence detection. J. King Saud. Univ. -Sci..

[6] Alshannaq A.F., Gibbons J.G., Lee M.-K., Han K.-H., Hong S.-B., Yu J.-H. (2018). Controlling aflatoxin contamination and propagation of Aspergillus flavus by a soy-fermenting Aspergillus oryzae strain. Sci. Rep..

[7] Awuor A.O., Yard E., Daniel J.H., Martin C., Bii C., Romoser A., Oyugi E., Elmore S., Amwayi S., Vulule J., Zitomer N.C., Rybak M.E., Phillips T.D., Montgomery J.M., Lewis L.S. (2017). Evaluation of the efficacy, acceptability and palatability of calcium montmorillonite clay used to reduce aflatoxin B1 dietary exposure in a crossover study in Kenya. Food Addit. Contam. Part A, Chem., Anal., Control, Expo. Risk Assess..

[8] Bennett J., Klich M. (2003). chotoxins. C lin. Microbiol. Rev..

[9] Bumbangi N.F., Muma J.B., Choongo K., Mukanga M., Velu M.R., Veldman F., Hatloy A., Mapatano M.A. (2016). Occurrence and factors associated with aflatoxin contamination of raw peanuts from Lusaka district’s markets, Zambia.. Food Control.

[10] Cotty P.J., Mellon J.E. (2006). Ecology of aflatoxin producing fungi and biocontrol of aflatoxin contamination. Mycotoxin Res..

[11] Deabes M., Al-Habib R. (2011). Toxigenic fungi and aflatoxin associated to nuts in Saudi Arabia. J. Am. Sci..

[12] Ding X., Wu L., Li P., Zhang Z., Zhou H., Bai Y., Chen X., Jiang J. (2015). Risk assessment on dietary exposure to aflatoxin B1 in post-harvest peanuts in the Yangtze River ecological region. Toxins.

[13] Elzupir A.O., Salih A.O.A., Suliman S.A., Adam A.A., Elhussein A.M. (2011). Aflatoxins in peanut butter in Khartoum State, Sudan. Mycotoxin Res..

[14] Eskola M., Kos G., Elliott C.T., Hajšlová J., Mayar S., Krska R. (2020). Worldwide contamination of food-crops with mycotoxins: Validity of the widely cited ‘FAO estimate’of 25%. Crit. Rev. Food Sci. Nutr..

[15] FAO (Food and Agriculture Organization). (2003). *No Worldwide Regulations for mycotoxins in food and feed 2003*.

[16] Gbashi, S., Madala, N.E., De Saeger, S., De Boevre, M., Adekoya, I., Adebo, O.A., & Njobeh, P.B. (2018). The socio-economic impact of mycotoxin contamination in Africa. *Fungi and Mycotoxins-Their Occurrence, Impact on Health and the Economy as Well as Pre-and Postharvest Management* Strategies (Ed. Njobeh, PB), 1–20.

[17] Jallow A., Xie H., Tang X., Qi Z., Li P. (2021). Worldwide aflatoxin contamination of agricultural products and foods: From occurrence to control. Compr. Rev. Food Sci. Food Saf..

[18] Jallow E., Jarju O.M., Mendy B., Dumevi R., Mendy W.F., Cham K. (2019). The trend of aflatoxin contamination levels in groundnuts from 2008-2018 in The Gambia. Lond. J. Res. Sci.: Nat. Form..

[19] Jedrzejczak R., Brulińska-Ostrowska E., Traczyk I. (2004). The activity of codex alimentarius commission FAO/WHO committee on methods of analysis and sampling. Rocz. Panstw. Zakl. Hig..

[20] Kamala A., Shirima C., Jani B., Bakari M., Sillo H., Rusibamayila N., De Saeger S., Kimanya M., Gong Y.Y., Simba A. (2018). Outbreak of an acute aflatoxicosis in Tanzania during 2016. World Mycotoxin J..

[21] Li Z., Cui J., Zhang X., Kang W. (2010). Aflatoxin G1 reduces the molecular expression of HLA-I, TAP-1 and LMP-2 of adult esophageal epithelial cells in vitro. Toxicol. Lett..

[22] Luttfullah G., Hussain A. (2011). Studies on contamination level of aflatoxins in some dried fruits and nuts of Pakistan. Food Control.

[23] Navale V., Vamkudoth K.R., Ajmera S., Dhuri V. (2021). Aspergillus derived mycotoxins in food and the environment: Prevalence, detection, and toxicity. Toxicol. Rep..

[24] Ostry V., Malir F., Toman J., Grosse Y. (2017). Mycotoxins as human carcinogens—the IARC Monographs classification.. Mycotoxin Res..

[25] Ráduly Z., Szabó L., Madar A., Pócsi I., Csernoch L. (2020). Toxicological and medical aspects of Aspergillus-derived mycotoxins entering the feed and food chain. Front. Microbiol..

[26] Rao V.K., Shilpa P., Girisham S., Reddy S.M. (2011). Incidence of mycotoxigenic penicillia in feeds of Andhra Pradesh, India. Int. J. Biotechnol. Mol. Biol. Res..

[27] Schwartzbord J.R., Brown D.L. (2015). Aflatoxin contamination in Haitian peanut products and maize and the safety of oil processed from contaminated peanuts. Food Control.

[28] SFDA (2019). *SFDA.FD 193:2019 Contaminants and toxins in food and feed*.

[29] Soares R.R.G., Ricelli A., Fanelli C., Caputo D., de Cesare G., Chu V., Aires-Barros M.R., Conde J.P. (2018). Advances, challenges and opportunities for point-of-need screening of mycotoxins in foods and feeds. Analyst.

[30] Sohrabi N., Taghizadeh M. (2018). Molecular identification of aflatoxigenic Aspergillus species in feedstuff samples. Curr. Med. Mycol..

[31] Suanthie Y., Cousin M.A., Woloshuk C.P. (2009). Multiplex real-time PCR for detection and quantification of mycotoxigenic Aspergillus, Penicillium and Fusarium. J. Stored Prod. Res..

[32] The American Peanut Council (APC). (2009). FOOD MANUFACTURING PRACTICES AND INDUSTRY BEST PRACTICES FOR PEANUT PRODUCT MANUFACTURERS.

[33] Udomkun P., Mutegi C., Wossen T., Atehnkeng J., Nabahungu N.L., Njukwe E., Vanlauwe B., Bandyopadhyay R. (2018). Occurrence of aflatoxin in agricultural produce from local markets in Burundi and Eastern Democratic Republic of Congo. Food Sci. Nutr..

[34] Waliyar F., Osiru M., Ntare B.R., Kumar K.V.K., Sudini H., Traore A., Diarra B. (2015). Post-harvest management of aflatoxin contamination in groundnut. World Mycotoxin J..

[35] Wu F. (2015). Global impacts of aflatoxin in maize: trade and human health. World Mycotoxin J..

[36] Yu J. (2012). Current understanding on aflatoxin biosynthesis and future perspective in reducing aflatoxin contamination. Toxins.

