# Supplementary material for: Occurrence of total aflatoxins (B1, B2, G1, G2) in commercial peanut and peanut butter from the Saudi market in the period from 2015 to 2020
Source: Toxicol Rep. 2024 Nov 18;13:101797. doi: 10.1016/j.toxrep.2024.101797 (PMC11667187; doi:10.1016/j.toxrep.2024.101797)
Supplement: Supplementary file 1 — Supplementary material [file mmc1.docx]

**Supplementary data**

**List of tables**

**Table 1.** Recoveries, LOD, LOQ and linearity for the analysis of Total Aflatoxins in Peanut

**Table 2:** The data of total aflatoxins in peanuts and peanuts butter samples from 2015-2020

**Table 3:** A survey of peanut and peanut butter contaminated with aflatoxins

**Table 1.** Recoveries, LOD, LOQ and linearity for the analysis of Total Aflatoxins in Peanut.

| **Analyte** | **Mean recoveries (%)** | **RSD%** | **LOD (µg/Kg)** | **LOQ (µg/Kg)** | **Linearity** |
| --- | --- | --- | --- | --- | --- |
| **Aflatoxin B1** | 107 | 5.23 | 0.38 | 1.26 | r2 = 0.999 |
| **Aflatoxin B2** | 105 | 5.57 | 0.10 | 0.33 | r2 = 0.999 |
| **Aflatoxin G1** | 108 | 5.68 | 0.20 | 0.69 | r2 = 0.999 |
| **Aflatoxin G2** | 83 | 7.25 | 0.10 | 0.33 | r2 = 0.999 |
| **Total Aflatoxin** | 104 | 5.51 | 0.77 | 2.59 | r2 = 0.999 |

**Table 2:** The data of total aflatoxins in peanuts and peanuts butter samples from 2015-2020


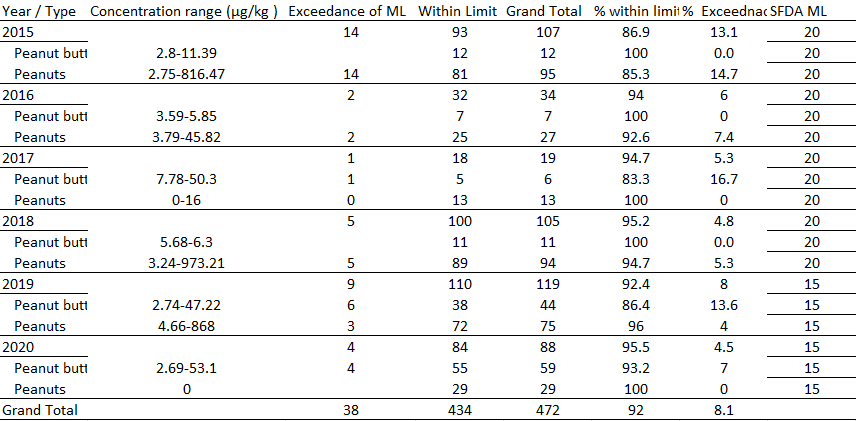


**Table 3:** A survey of peanut and peanut butter contaminated with aflatoxins

| Country | Food matrices | Sample size | Concentration range (ug/kg) | Reference |
| --- | --- | --- | --- | --- |
| Gambia | peanut | 1168 | 112-8.55 | (E. Jallow et al., 2019) |
| Zambia | Raw peanut | 92 | 48.67 –0.014 | (Bumbangi et al., 2016) |
| Haiti | Peanut | 21 | 787-2.0 | (Schwartzbord & Brown, 2015) |
|  | Peanut butter | 32 | 2720-2.0 |  |
| Saudi Arabia | peanut | 5 | 90-11 | (Deabes & Al-Habib, 2011) |
| Pakistan | Peanut with shell | 10 | 14.5-1.5 | (Luttfullah & Hussain, 2011) |
|  | Peanut without shell | 10 | 12.8-0.7 |  |
| Sudan | Peanut butter | 43 | 853-26.7 | (Elzupir et al., 2011) |
| China | peanut | 2983 | 113.50-0.16 | (Ding et al., 2015) |

**Supplementary data**

**List of Figures**

**Figure 1**. The chromatogram of separated to total Aflatoxin by HPLC-FLD

**Figure 2.** The percentage of exceedance limit of the samples from different countries;2015-2020

**Figure 3.** The exceedance of limit and total samples from 2015-2020

**Figure 4**. Comparing the exceedance of limit samples from 2015-2018 and 2019-2020

**Figure 1.** The chromatogram of separated to total Aflatoxin by HPLC-FLD


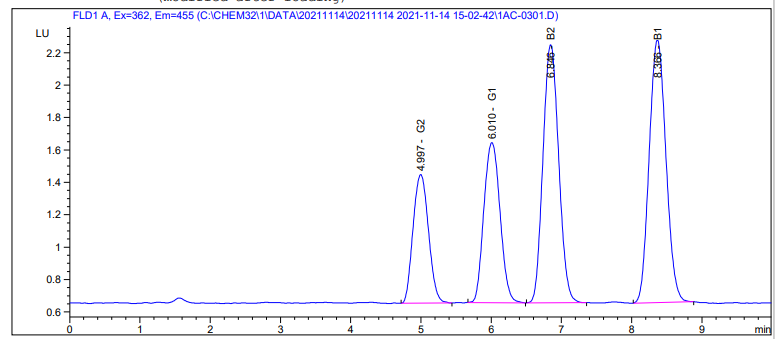


**Figure2.** The percentage of exceedance limit of the samples from different countries;2015-2020

**Figure 3.** the exceedance of limit and total samples from 2015-2020


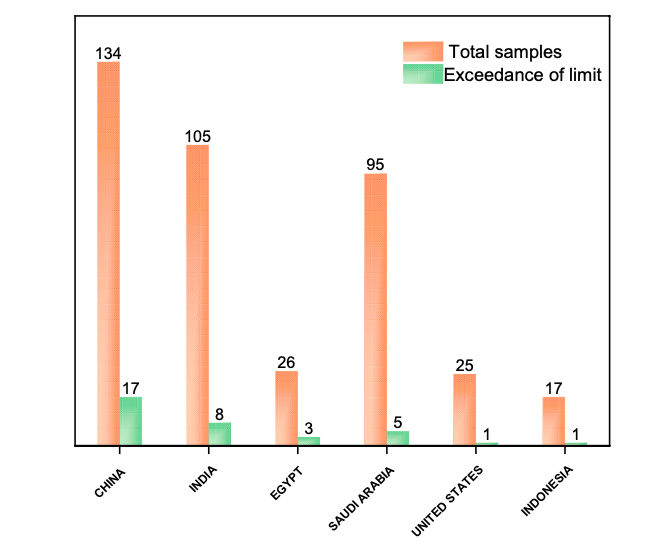


**Figure 4**. Comparing the exceedance of limit samples from 2015-2018 and 2019-2020
